# Editorial: The role of post-translational modifications in cancer biology

**DOI:** 10.3389/fonc.2024.1470173

**Published:** 2024-08-28

**Authors:** Swati Srivastava, Anurag Tripathi, Arun Kumar Trivedi

**Affiliations:** ^1^ Division of Cancer Biology, CSIR-Central Drug Research Institute, Lucknow, Uttar Pradesh, India; ^2^ Food Toxicology Group, CSIR- Indian Institute of Toxicology Research, Lucknow, India; ^3^ Academy of Scientific and Innovative Research (AcSIR), Ghaziabad, India

**Keywords:** post translational modification (PTM), ubiquitination, phosphorylation, cancer, acetylation

## Introduction

This editorial presents articles published on the Research Topic “The role of post-translational modifications in cancer biology” in the journal “Frontiers in Oncology”. It aimed to explore post-translational modifications (PTMs) of key proteins and their association with cancer pathogenesis. Various articles in this Research Topic highlight PTMs of key oncogenic factors and provide a deeper insight into the basic, translational, and clinical implications of protein modifications.

The article by Kellete et al. demonstrated the most frequently reported PTM, phosphorylation, which is often involved in multiple cellular signaling pathways. They showed that the phosphorylation of focal adhesion kinase (FAK) protein at pY397 is responsible for its nucleolar translocation, activation, and accumulation in thyroid cancers. In addition, phosphorylated FAK (pY397) also regulates 60S ribosomal subunit biogenesis by co-localizing with other nucleolar proteins.

Ubiquitination, another PTM that plays a key role in maintaining protein homeostasis, has also been implicated in tumorigenesis. Ubiquitin–proteasome system (UPS) is a multistep process involving the sequential action of various enzymes, leading to proteasome-mediated protein degradation. This biological process is mediated by E1, E2, and E3 enzymes that work sequentially and carry out ubiquitin activation, conjugation, and ligation, respectively. Abnormalities in any of these enzymes may lead to dysregulated protein turnover resulting in disease pathology. E1 enzymes activate the ubiquitin molecules followed by the conjugation of activated ubiquitins by E2 enzymes. E3 enzymes, also known as E3 ubiquitin ligases, add the ubiquitin molecules to the target proteins leading to their polyubiquitination and subsequent degradation by the proteasome machinery (1, 2). Deubiquitinases (DUBs), another class of enzymes, are also an essential component of the UPS that removes the ubiquitin chains from protein substrates, thus reversing the ubiquitination process.

Several articles in this Research Topic addressed the involvement of various enzymes in the ubiquitination process. Guo et al. showed that the ubiquitin-conjugating enzymes E2S (UBE2S) and E2C (UBE2C) are upregulated in breast cancer patients and are associated with poor survival. Interestingly, UBE2S and UBE2C target a key tumor suppressor, Numb, which is also lowly expressed in patients with breast cancer. They argue that the combination of UBE2S/UBE2C and Numb could potentially serve as novel biomarkers for breast cancer. The report by Liu et al. highlighted the role of E3 enzymes in cancer progression. Through gene expression omnibus (GEO) analysis, they identified the potential really interesting new gene (RING) finger protein family (RNF) members that form the majority of ubiquitin ligases in case of non-small cell lung cancer (NSCLC). They showed that Benzo[a]pyrene (BaP), an environmental carcinogen, suppresses the expression of RNF182 in NSCLC. Mechanistically, BaP promotes abnormal methylation of RNF182 gene, leading to its suppression resulting in the progression of lung cancer. It is intriguing to see the epigenetic regulation of E3 enzymes and its consequences in tumor progression.

Other reports highlighted the role of DUBs in cancer progression. Timani et al. showed that Tip110 activates NF-κB signaling by targeting IκBα stability through ubiquitin-specific peptidase (USP15). With various intricate assays, they showed that Tip110 potentiates IκBα phosphorylation, leading to its degradation and hence, increase in NF-κB activity. On the other hand, authors also showed that IκBα is a substrate of USP15 that stabilizes IκBα and thus inhibits NF-κB activation. They demonstrated that Tip110 also interacts with USP15 and affects its nuclear localization. It seems that fine-tuning in the triad of Tip110-USP15-IκBα is crucial for the NF-κB signaling. Another oncogenic deubiquitinase in pancreatic cancer was highlighted by Liu et al. They discovered a novel deubiquitinase MINDY2 (a member of the motif interacting with Ub-containing novel DUB family). Pancreatic cancer is one of the deadliest cancers since patients with pancreatic cancer usually present with advanced-stage cancer at the time of diagnosis. They showed that MINDY2 expression is elevated in cancer tissues as compared to their adjacent normal and correlates with poor prognosis, distant metastasis, and angiogenesis of cancer cells. They further showed that MINDY2 interacts with ACTN4 and stabilizes it through deubiquitination. This stabilized ACTN4 stimulates the PI3K/AKT/mTOR pathway leading to the proliferation and invasive metastasis of pancreatic cancers. Similarly, Ma et al. showed that levels of UCHL3, yet another deubiquitinase, is significantly elevated in hepatocellular carcinoma and is associated with poor prognosis. Interestingly, similar to MINDY2-ACTN4 crosstalk, UCHL3 stabilizes another structural protein Vimentin, leading to metastasis.

Moreover, Zhang et al. identified Dishevelled, Egl-10 and Pleckstrin (DEP) domain-containing protein 1B (DEPDC1B) to have an oncogenic role in cholangiocarcinoma. Unlike the above studies, DEPDC1B was shown to stabilize CDK1 by inhibiting SMURF1-mediated CDK1 ubiquitination. DEPDC1B was shown to interfere in the protein–protein interaction between Smurf1 and CDK1. Notably, Smurf1 is an E3 ubiquitin ligase of CDK1. Other than proteasome-mediated degradation, lysosomal degradation pathway is also one of the key mechanisms to regulate protein turnover. Si et al. demonstrated that coiled-coil domain containing 102B (CCDC102B) was significantly upregulated in metastatic lesions in lymph nodes compared to matched primary breast tumors. RACK1 promoted CCDC102B lysosomal degradation through chaperone-mediated autophagy. The aggressive behavior of CCDC102B in breast cancer cells could be reversed by the expression of RACK1. The article by Wang et al. discussed yet another important PTM, acetylation. It is intriguing to see the role of protein acetylation not only in subcellular translocation but also in the activation of a key oncogenic signaling pathway. They showed that Wnt ligand stimulation leads to SIRT4 translocation from the mitochondria to the cytoplasm where it deacetylates Axin1-K147, resulting in the reduced assembly of β-TrCP to the destruction complex and thus, leading to β-catenin nuclear translocation and accumulation. Finally, Dutta and Jain summarized glimpses of different PTMs in cancer pathology in a minireview where they not only highlighted the conventional PTMs but also discussed several less-reported PTMs such as monoaminylation, crotonylation, propionylation, butyrylation, and lactylation. Summarily, the findings of the collective articles presented in this editorial highlight the role of distinct PTMs of various key proteins that impact their functionality and contribute to cancer pathogenesis.
